# Yoga interventions and randomized controlled trials: key issues and the centered impact on sedentary lifestyle-associated cardiometabolic disorders

**DOI:** 10.3389/fcdhc.2026.1805160

**Published:** 2026-04-24

**Authors:** Rakhi Radhamani, Shyam Diwakar

**Affiliations:** 1 Amrita Mind Brain Center, Amrita Vishwa Vidyapeetham, Kollam, Kerala, India; 2 Department of Electronics and Communication Engineering, Amrita Vishwa Vidyapeetham, Kollam, Kerala, India

**Keywords:** cardiometabolic disorders, non-communicable diseases, public health, statistical heterogeneity, yoga training

## Abstract

**Introduction:**

Yoga training has been shown to reduce health risks associated with a sedentary lifestyle. This meta-analytical approach systematically assesses the impact of Hatha yoga on non-communicable diseases (NCDs) and cardiometabolic disorders linked to sedentary lifestyles, including type II diabetes, hypertension, and cardiovascular diseases (CVDs)—the key contributors to morbidity and mortality in India.

**Methods:**

Eighteen randomized controlled trials (RCTs) were selected from database searches (PubMed, Cochrane Central, Google Scholar, and Scopus) and involved patients diagnosed with NCDs. The meta-analysis included 644 participants for studies on diastolic blood pressure (DBP), 592 for those on systolic blood pressure (SBP), 1,387 for fasting blood glucose (FBG), 1,243 for postprandial blood sugar (PPBS), 963 for total cholesterol (TC), and 772 for low-density lipoprotein (LDL) levels.

**Results:**

Statistical analysis of the RCTs indicated that yoga training improved stress-related physiological responses, reduced the risk of hypertension, was a complementary intervention for diabetes management, and regulated lipid biomarkers associated with CVDs compared to control conditions (usual care or physical exercise). The variability between the studies and the resultant heterogeneity across the outcome measures may influence the precision of the pooled estimates. The observed effects reported in this study were indicative trends of yoga interventions in managing cardiometabolic disease-associated risk factors.

**Discussion:**

Yoga as a potential alternative and complementary therapeutic approach in mitigating risks from sedentary lifestyle-driven NCDs, particularly T2DM, HTN, and CVDs. We also report that heterogeneity among studies must be addressed by delivering standardized yoga protocols and yoga training strategies uniformly across diverse populations in future studies focusing on cardiometabolic outcome measures and yoga practices.

## 1 Introduction

A sedentary lifestyle has become a global health issue due to inadequate physical activity. An insufficiently active lifestyle is defined as difficulty performing approximately 2 hours of moderate-intensity activity per week, such as leisure time, household activities, sports, or regular exercise (1). Many factors, including demography, psychosocial impacts, behavioral intentions, and social factors, contribute to sedentary activities (2). The long-term use of television (TV) digital technology, and increased screen time associated with online games and use of social networking sites, were major determinants of sedentary behaviors (3). Prolonged sitting hours at work, job stress, and public socialization without any physical activity are other common factors leading to this lifestyle (4). According to World Health Organization (WHO), sedentary lifestyles associated with non-communicable diseases (NCDs) have emerged as a public health threat at a global scale and are leading causes of death worldwide (5–7). Sedentary lifestyles has been linked to elevated risk factors associated with diabetes mellitus (DM), cardiovascular disease (CVD), hypertension (HTN), and different types of cancer (8).

**Figure 1:**
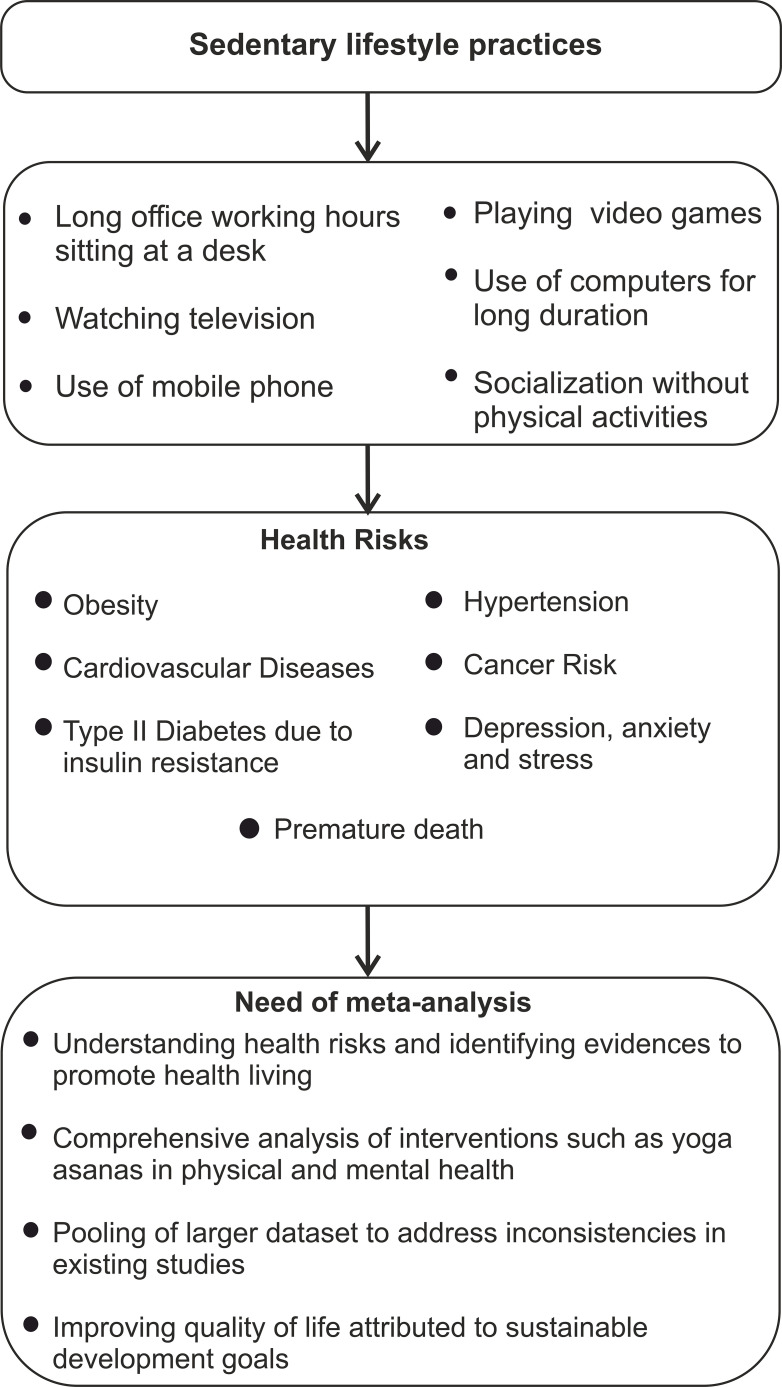
Graphical abstraction of sedentary lifestyle, associated risk factors, and the perspectives of meta-analysis.

Furthermore, it has been linked to metabolic dysfunctions, including elevated plasma triglycerides, altered cholesterol levels mainly high-density lipoprotein (HDL), and impaired insulin sensitivity (9). It has been associated with osteoporosis, associated with bone mineral density, and musculoskeletal conditions that contribute to chronic knee pain (10). Diabetic neuropathy (DN) is another chronic complication, like cardiovascular complications, that occurs due to poor glycemic control, leading to peripheral nerve dysfunction in patients with diabetes, reducing the patient’s exercise ability (11). A cross-sectional fMRI study indicated similar cognitive profiles and variations in brain connectivity patterns among individuals with different extent of physical activity (12). Another study reported that yoga and meditation practices as lifestyle interventions significantly reduced cellular aging in a healthy population (13). Diabetic retinopathy (DR) is another eye-threatening microvascular consequence of diabetes mellitus causing irreversible blindness (14). Even though these complications were well-known, the relationship between cognitive functions and sedentary behavior remains unclear. Understanding how sedentary life and associated behavioral patterns increase the risk of cardiometabolic diseases remains uncovered. (15). A known approach to combat these conditions is to incorporate physical activities into daily activities to promote overall health (16). The global action plan on physical activity 2018–2030 by WHO had highlighted yoga training as a cost-effective and feasible physical activity for individuals of all ages for disease management (17) related to NCDs for a sustainable approach to overall well-being (18, 19) and adequate prevention and management strategies (5, 19, 20) (**Figure 1**).

Empirical analysis indicated interest of scientific community in providing personalized and holistic approaches as treatment regimens (21). Yoga-based physical exercises play a pivotal role in combating stress and inflammatory conditions that are common in NCDs (22). Studies have focused on integrating yoga with modern therapeutic strategies to ensure effective treatment in the public health domain (23). The goal of this study was to evaluate research studies that employed multifaceted approaches of integrating yoga into daily living (ADL) (24, 25). A major focus was to analyze how yoga training influences mind-body relationships (26, 27) and review existing yoga therapy protocols that may complement modern medicine (21, 28), for preventing and managing cardiometabolic diseases. This analysis focuses on how yoga protocols were designed, the study population, components of yoga, yoga delivery methods, intervention fidelity over time, which varied across studies and contributed to overall heterogeneity. Understanding these factors may aid in developing standardized yoga protocols to study the underlying neuroscience for interpreting neural correlates (29, 30) and associated cognitive functions. Research has indicated that yoga modulates neurotransmitter levels, such as elevating gamma-aminobutyric acid (GABA) levels, thereby attenuating stress, improved sleep regulation, homeostasis, improving cognitive performance, emotional regulation, and self-awareness (31). In addition to these, yoga practices have an important role in improving gait, muscular strength, and flexibility in supporting the integration of yoga for ADLs (32).

Evidence from meta-analysis indicated that yoga interventions were associated with improvements in the psychological health in cancer patients, particularly by alleviating stress and anxiety, compared with those nonintervention group (33). Yoga interventions have been reported as a treatment measure for reducing lower back pain and its complications among regular practitioners compared to usual care or therapeutic measures (34). An analysis on managing type-II diabetes indicated that daily practice of yoga benefitted glycemic management. (35). Related studies have reported evidence of clinically relevant aspects of yoga regimens on managing cardiovascular risk factors in both healthy subjects and high-risk population (36). Studies have also reported the importance of yoga practices for controlling hypertension (37, 38). Apart from inconsistent intervention protocols, heterogeneity among studies was a common observation that may have impacted the quality of many meta-analysis studies. A substantial increase in the ratio of people who suffered acute coronavirus infection and developed post-COVID-19 sequelae, reported that yoga intervention was a choice for mitigating associated broader public health issues (39). Another study among university students highlighted that following a 12-week yoga intervention, a notable reduction in the perceived stress and anxiety scores along with improved emotional well-being in students was observed (40). A study credited the common yoga protocol (CYP) played a role in attributing regular yoga practice as a strategy to prevent cardiovascular diseases (41).

Interpreting the efficacy of such interventions for overall well-being demands addressing gaps in existing studies. The key question to address is how daily yoga practice with a specific frequency and duration can bring lifestyle modifications in sedentary individuals. The empirical studies of this meta-analysis highlighted the need for strategizing a multitude of unanswered questions on daily yoga routines for improving quality of life by bringing sustainable development goals in health for future perspectives. The WHO had reported ~2 million deaths yearly were connected to physical inactivity and sedentary behaviors were a primary cause of death and disability across the world (42). The majority of passive lifestyle-associated NCD deaths were reported across developing countries with moderate income levels, with India also significantly affected (43), which may be attributed to rapid urbanization and associated epidemiological health transitions (44). India being ascribed the diabetes capital of the world, this meta-analysis study focused on cardiometabolic assessments related to type II diabetes, cardiovascular diseases, and hypertension, as they are closely interlinked because of similar risk factors and biomarkers associated (45–59) (**Table 1**) with 2‐ to 4‐fold higher mortality than other diseases. The current study also focuses on delivering an analysis of the reported impact of yoga practices on reducing the symptoms associated with NCDs, leading to cardiometabolic risks that are closely related to sedentary lifestyle practices. This work analyzes studies related to randomized control trials that used yoga interventions to focus on the following questions:

**Table 1:** Key biomarkers and their clinical implications in cardiometabolic disease risk factors.

Biomarker	Risk Factors connected to non-communicable diseases
Blood Pressure (BP)	Systolic and diastolic blood pressure directly contributes to increased risk of adverse cardiovascular events (49, 53).
Total Cholesterol (TC)	The main cause of coronary atherosclerosis (52)
Low-density lipoproteins (LDL-C)	Considered a critical contributor in the progressive evolution of atherosclerosis that is associated with cardiovascular disease and plaque formation (58)
High-Density Lipoprotein (HDL-C)	Elevated HDL-C levels are directly associated with a reduced risk of coronary artery disease (46).
Heart Rate (HR)	Accelerating heart rate is related to progressive cardiovascular disease risk (55).
Triglycerides (TG)	An increase in serum triglycerides (TG) is linked directly to the increase in cardiovascular disease risk. The exact metabolic connection between TG and CVD risk remains unanswered (45).
Fasting Blood Glucose (FBG)	Diabetes is considered a major factor for cardiovascular disease with reduced insulin sensitivity, pancreatic beta cell dysfunction, and atherosclerosis promotion (59).
Postprandial Blood Glucose (PPBS)	Predictor for cardiovascular events and death in Type II Diabetes (47, 51)
Hemoglobin A1C (HbA1c)	High blood glucose levels predict a greater risk of CVD in people with or without diabetes (50).
C-reactive protein (CRP)	A novel inflammatory marker that plays a role in predicting the progression of cardiovascular events in individuals with and without coronary heart disease (57).
Interleukin-6 (IL-6)	Increase in IL-6 levels activate atherothrombotic events and have a potential causal role in atherosclerosis (56).
Nitric Oxide (NO)	Protective role against the onset of cardiovascular disease events. Reduction in the bioavailability of NO central factor for cardiovascular disease due to endothelial dysfunction (48, 54).

1. Does yoga training positively impact sedentary lifestyle-induced cardiometabolic risk factors associated with cardiovascular health, blood pressure, and type II diabetes?
2. What do outcome and effect analysis indicate as the overall influence on the quality of life and well-being of individuals associated with sedentary habits who may have integrated yoga training?

The literature analysis shows that limited research has investigated yoga’s impact on sedentary life-associated NCDs, particularly cardiometabolic risk factors. A comprehensive analysis of yoga interventions with a focus on RCTSs and cardiometabolic risk factors has not been conducted, especially in the context of India where lifestyle-related NCDs are rising at an alarming rate.

## 2 Methods

This study’s systematic analysis was conducted in accordance with the standards presented in the Preferred Reporting Items for Systematic Reviews and Meta-Analyses (PRISMA) guidelines. The authors conducted a preliminary search for published systematic reviews or protocols related to the topic and have not found any published protocol related to short-term yoga training on sedentary lifestyle-associated cardiometabolic disorders. This study protocol did not foresee any known biases in the methodological approaches.

### 2.1 Selection criteria

The eligibility criteria followed the well-known structure, including Participants, Intervention, Control, Outcome, and Study Design (PICOS). The inclusion criteria comprised of the following constructions:

1. The selected studies were conducted on participants with known type II diabetes mellitus, cardiovascular diseases, and blood pressure (P).
2. The studies that assessed yoga intervention, such as asana, pranayama practices, or meditation, were compared with those of usual care, control, or placebo groups (I and C).
3. Studies that evaluated and compared at least one of the predefined primary or secondary outcome variables were included(O).
4. A randomized controlled trial (RCT) with an experimental design was included (S).
5. Full-text articles written in English reported sedentary lifestyle-associated factors including cardiovascular complications, type II diabetes mellitus, and hypertension.
6. Studies based in India.

The exclusion criteria were systematic reviews, clinical trials, meta-analyses, or abstract-only articles. Studies published prior to 2000 were excluded, as the disease prevalence or conditions may have shifted over time, and the older studies were less relevant to the current public health questions addressed in this meta-analysis. Although research on yoga studies existed before 2000, articles often lacked rigorous methodology, such as detailed descriptions of yoga protocols and interventions required for evidence-based medicine, which makes it difficult to replicate the protocol for extended studies. The variation in yoga practice, duration, intensity, and delivery methods made the comparison across studies difficult. This recommended the need for high quality RCTs for reducing potential biases. This meta-analysis initially searched for studies conducted globally, however, structured intervention protocols based on traditional yogic frameworks have been consistently implemented in Indian integrative medicine departments compared to many other countries. We also noted that RCTs evaluating the role of yoga interventions are not comparable globally, as the protocols for yoga practice vary considerably across countries (60–62).

Yoga has been reported employed as an integral component of healthcare in India and a part of Indian traditional medicine; the present meta-analysis focused on including more recent studies originating since 2000 within India as the geographical location to minimize variability in practice frameworks and intervention strategies. Furthermore, the worldwide prevalence of lifestyle disorders, particularly Type-2 Diabetes Mellitus (T2DM), and associated cardiometabolic disorders has increased dramatically worldwide over the past two decades and has reached near-pandemic levels. Although the research is increasingly in conducted many countries, significantly half of published randomized controlled trials (RCTs) on yoga were from India (63).

### 2.2 Search strategy

A systematic and comprehensive electronic search was performed using PubMed, Cochrane Central, Google Scholar, and Scopus databases to identify studies reporting outcomes of yoga training in reducing noncommunicable diseases and improving cardiometabolic activity. The search period was from January 1, 2000, to May 2024. To extract relevant studies, the literature search was designed based on predefined search terms such as “yoga” or “yoga for sedentary lifestyle” with filters limited to randomized controlled trials (RCTs).

**Figure 2:**
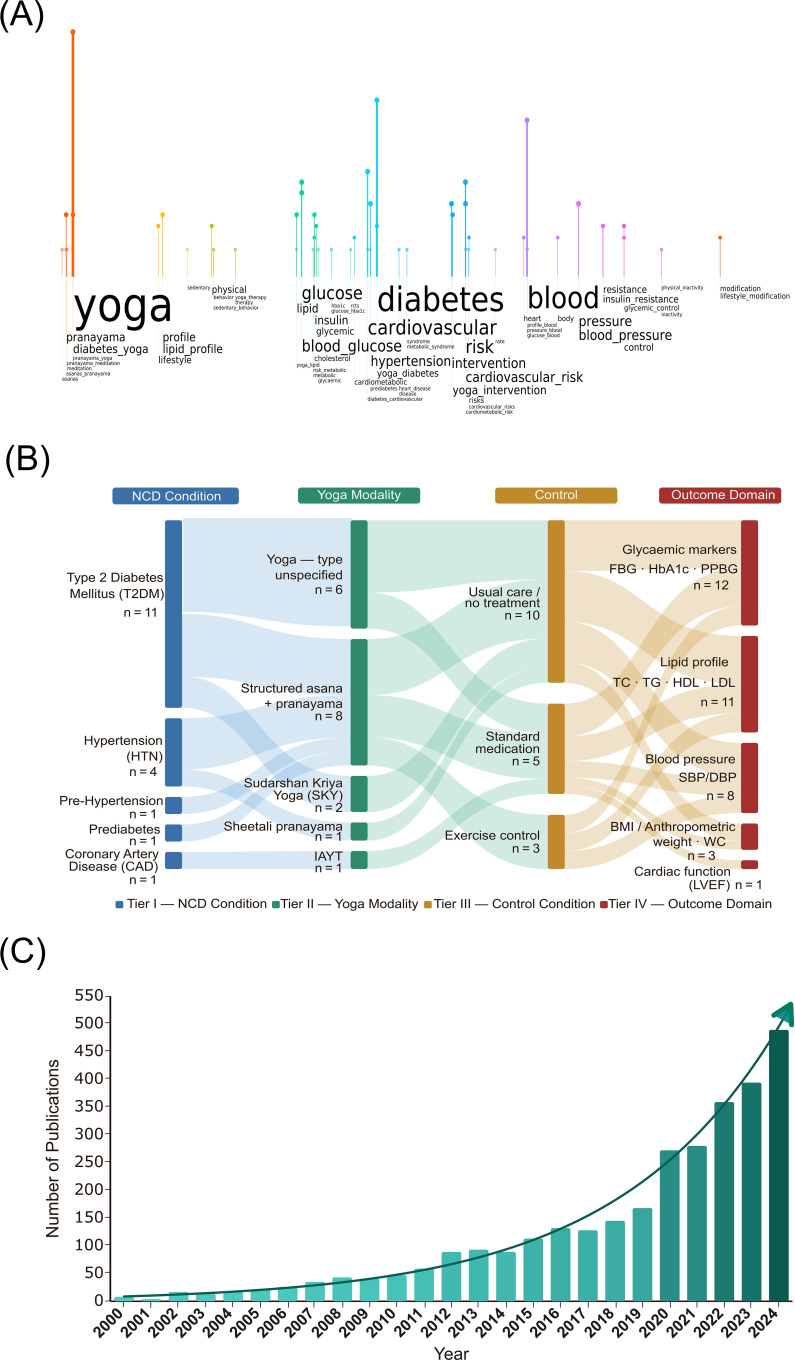
Multidimensional overview of research trends, depicting yoga-based intervention strategies in the management of cardiometabolic-associated non-communicable diseases. **(A)** Rain-cloud plot of the keyword occurrence map, showing the strategies in the included studies. The size and clustering of key terms indicate the frequency and interrelationships among them, emphasizing the role of yoga interventions in cardiometabolic outcomes. **(B)** Sankey diagram representing the flow of evidence, NCD condition, yoga modalities, control conditions, and outcome domains in the included studies. **(C)** Temporal trend of publications from 2000 related to yoga, diabetes, and cardiovascular diseases in India. The upward trajectory illustrates the progressive increase in research output over time and scientific interest in including yoga as a complementary intervention for managing complications in NCD populations.

In addition to keyword searches, the search strategy also included relevant MeSH terms to ensure comprehensive coverage of the literature. Searches were also performed for “Yoga”, “Yoga and cardiovascular diseases”, “Yoga and hypertension” and “Yoga and type II diabetes” as subject terms. In addition, the study also employed a snowballing approach and cross-checking the reference sections of the included articles to locate additional studies of potential relevance. The authors performed an independent screening of the articles as per the predefined search strategy. Any conflicts or disagreements were addressed through an open-ended discussion between the authors. Duplicates were removed from the Mendeley database. In the initial literature search, 415 articles were retrieved according to the search criteria. After excluding duplicates and ineligible publications, 42 unique full-text records were identified. From this list, 18 RCTs (64–81) that satisfied the criteria for inclusion were added in the present systematic review (**Table 2**) and eliminated the ineligible studies (82–90). The distribution of studies across intervention types and outcome, and study characteristics, frequency distribution of keyword occurrences across the included studies and the temporal analysis in the number of publications from 2000 was analyzed (**Figure 2**).

**Table 2:** Characteristics of the studies selected for meta-analysis. Data has been retrieved by a random search of the existing literature.

Study Reference	NCD condition connecting to sedentary lifestyle	Subject information	Duration of yoga intervention	Analysis of intervention	Control condition	Outcome measurement
(72)	HTN	N=33, gender not revealed	11 weeks	Yoga Duration: 2hrs/week Type of yoga not mentioned	Usual care	Blood pressure, heart rate
(74)	Pre- HTN or HTN	N = 103; 33.3% female.	8 weeks	30–45 min yoga session for 5 days per week	Exercise (4 × 50–60 min/week) 2. Reduce in sodium intake 3. Usual care	Blood pressure
(73)	T2DM	N = 277; 31.4% female;	9 months	Yoga 5 × 1hour/week for 12 weeks + 1 × 2 - hours/week and 1 hour daily home practice for 9 months Type of yoga not mentioned	Exercise 5 × 60 min/week for 12 weeks + 1 × 120 min/ week and 1 h daily home 9 months duration	Total cholesterol, triglycerides, HDL, LDL, VLDL, fasting blood glucose, HbA1c
(75)	T2DM	N = 100; 48% female;	3 months	Yoga 7 × 1 hour/week Type of yoga not mentioned	Usual care	total cholesterol, HDL, LDL, triglycerides
(80)	T2DM	N = 60; 36.8% female;	12 weeks	Yoga 6 × 45–60 min/week	Usual care	Total cholesterol, HDL, LDL, triglycerides, fasting blood glucose, HbA1c
(78)	HTN	N= 100 gender not revealed	3 month	Sheetali pranayama	Usual care	Blood pressure and HRV
(66)	T2DM	N= 40 gender not revealed	3 months	15 asanas and 3 pranayama sessions, 75–90 minutes, over a 3-month duration. Type of yoga not mentioned	Nonaerobic ex + walking	SBP, DBP, BMI, WC etc.
(76)	Coronary artery disease	N= 104 gender not revealed	Yoga sessions were held three times weekly for 12 weeks.	IAYT classes	standard care	left ventricular ejection fraction
(77)	T2DM	N= 124 All-female subjects	3 months	Yogic postures and breathing exercises an hour, 2 days/week	Standard care	Fasting plasma glucose
(64)	Pre- HTN	N=102 gender not revealed	6 months	Different types of asanas were mentioned, for example Swastikasana, and Vajrasana	Standard care	blood pressure and weight monitoring and quality of life questionnaire
(79)	HTN	Volunteers (20–60 years) N=100 gender not revealed	12 weeks of intervention	Different types of asanas were mentioned	Usual care	blood pressure
(69)	Prediabetes	N= 184 26.6 % men	60-minute yoga sessions, conducted five days a week for 12 weeks.	Different types of asanas mentioned	Control condition following standard medication	FBG, PPBG, HbA1c, Lipids
(70)	T2DM	N= 38 42% men Drops out reported	75-minute yoga sessions per day, performed three to six days per week, for a total duration of eight weeks.	Standing asana, Supine asana, Prone asana, Sitting asana, Relaxation Shavasana, Chanting ‘OM’ monosyllables	30-min daily walking, 3–6 sessions per week over 8 weeks.	FBG, PPBG, HbA1c, BP, Lipids
(81)	T2DM	Yoga – M/F 93/57, Age 50.8 ± 8.3 Control – M/F 103/47, Age 52.8 ± 7.0 65.3 % men N= 300	50-min daily yoga, 5 sessions/week for 12 weeks	Different types of asanas mentioned Savasana-Relaxation	Control group received standard medication	FPG, PPBG, HbA1c, Lipids
(71)	T2DM	N=20 Female subjects	3 months	Suryanamaskara + asana + pranayama + meditation/35–55 min/3 months	Usual care	TC, TG, LDL, HDL
(65)	T2DM	N=80 All male subjects	Yoga 40 min/day, 5 days/week for 12 weeks with medication	yoga therapy that included asana and pranayama practice for 12 weeks	Control group following standard medication	FBG, BP, Insulin, BMI, Lipids
(67)	T2DM	N = 120; 38.9% female;	3 days/3 months	Duration: 3 days 12-hour course Sudarshan Kriya Yoga	Usual care	FBG, HbA1c
(68)	T2DM	N= 120; gender not revealed	3 days/6 months	Sudarshan Kriya Yoga	Usual care	Fasting blood glucose, HbA1c

**Figure 3:**
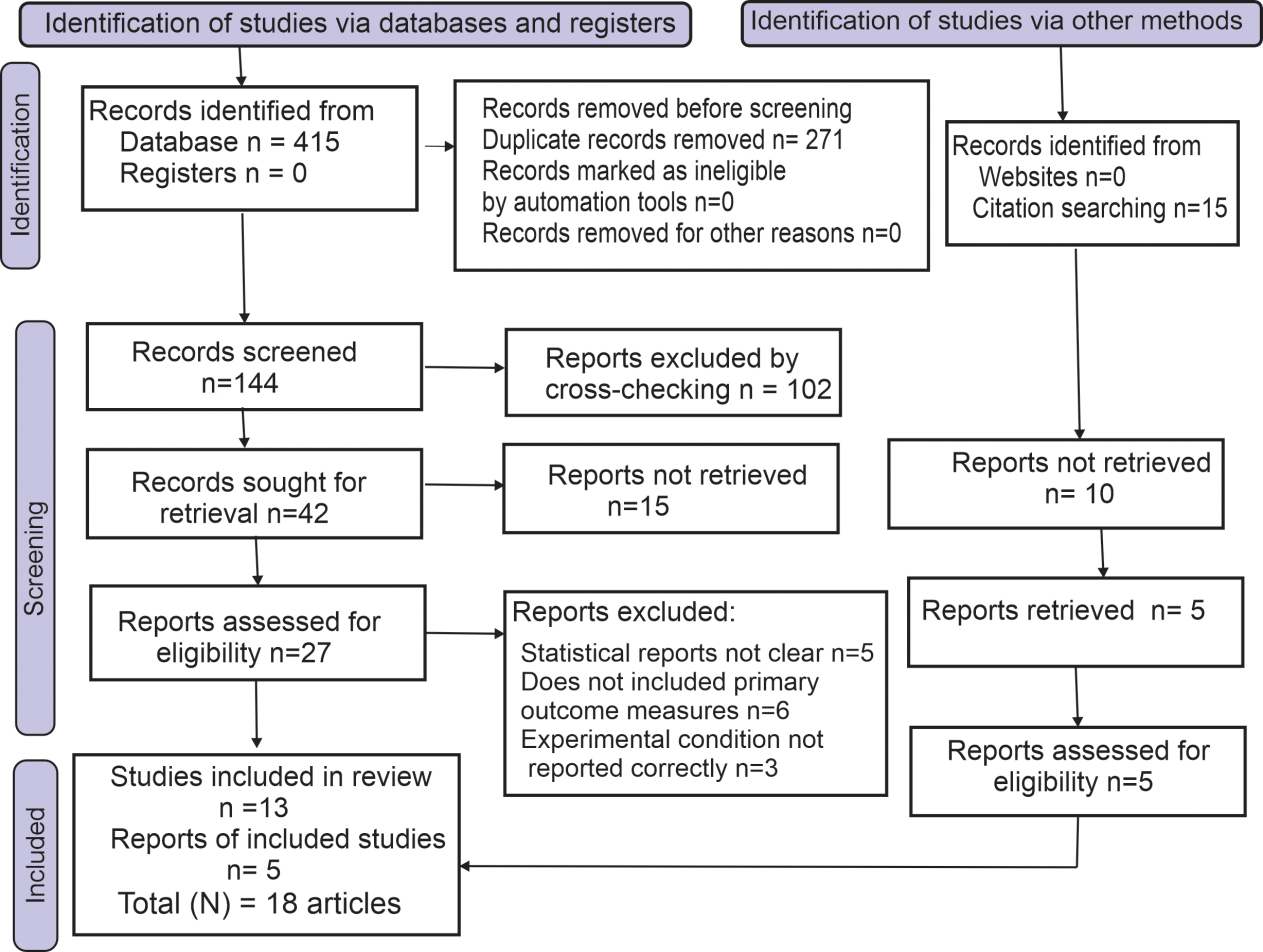
Database search and selection of studies according to the PRISMA 2020 guidelines. The flowchart depicts the process undertaken for the present meta-analysis review based on the systematic inclusion and exclusion strategy.

The PRISMA study selection flow diagram is presented in **Figure 3**. The included studies on yoga and lifestyle disorders reported their effects on cardiometabolic health, hypertension, and cardiovascular risk factors related to type II diabetes and hypertension (**Supplementary File** PRISMA checklist, **Supplementary Table 1**). These primary and secondary outcomes were collectively categorized within the noncommunicable diseases.

### 2.3 Data extraction and management

The authors independently collected information on the study location, year, participant characteristics, duration, sample size, intervention and control details, and outcome assessments. All the discrepancies were discussed until a general agreement was reached. Random effects were employed in the meta-analytic review to assess the heterogeneity across the yoga intervention study groups. Statistical heterogeneity evaluations were performed with the I^2^ statistics, with I^2^ > 50% considered indicative of substantial heterogeneity. Tau^2^ represents the estimated standard deviation of the effects reported across the included studies. For heterogeneity, a statistically significant level of P < 0.05 was defined to determine the significance of the reported analyses. Subgroup analysis was performed to further explore the sources of heterogeneity among the included studies followed by a leave-one-out sensitivity analysis to characterize the influence of individual studies on the pooled estimate.

### 2.4 Risk of bias assessment in individual studies

To evaluate potential biases, the Cochrane Collaboration’s modified tool (91) was employed to assess the included studies. The assessment tool addresses the key potential biases: random sequence generation and allocation concealment (selection bias), blinding of participants and personnel (performance bias), selective outcome reporting (reporting bias), blinding of outcome assessors (detection bias), incomplete outcome data (attrition bias) and other important sources of bias. The risk of bias was assessed for each domain is classified as low, unclear, or high. Disagreements were discussed by the authors until consensus was achieved and marked as resolved. The overall risk of bias in each domain for individual studies was categorized as low, high, or unclear. A bibliometric study with the key term yoga was also performed to examine the trends of contributions of different countries to yoga research.

### 2.5 Evaluating the certainty of evidence in selected studies

To assess the quality of evidence across the included studies, the Grading of Recommendations Assessment, Development and Evaluation (GRADE) framework was utilized (https://www.gradepro.org/). This approach estimated the certainty evidence biases apart from individual assessment of studies and correlated to high, moderate, low, and very low levels of certainty based on accuracy and biases in the observed results (92).

## 3 Results

No significant adverse effects were reported in any of the included studies.

### 3.1 Intervention studies confirm the risk of bias and selective outcome reporting

**Figure 4:**
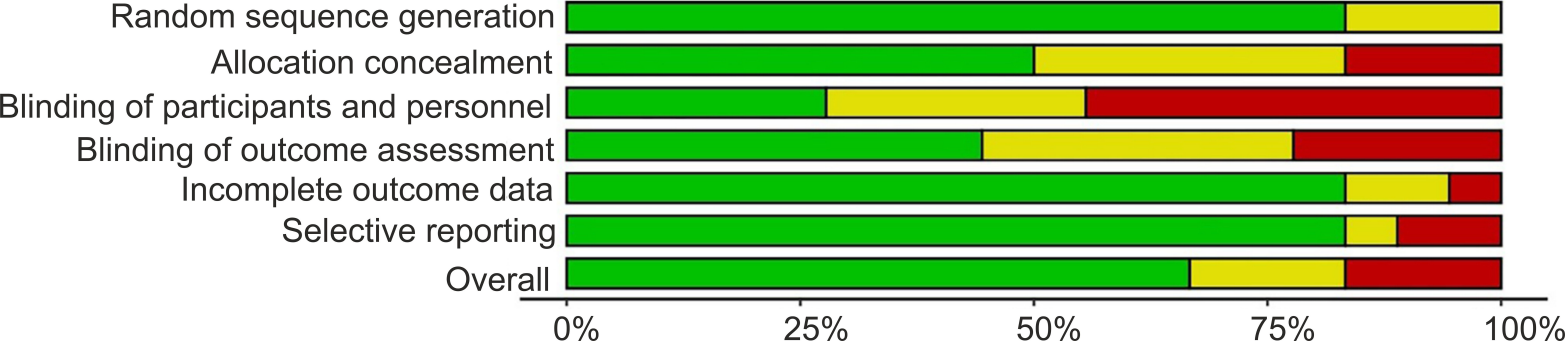
Overview of the risk of bias assessment of the studies selected for the meta-analysis. In the weighted bar plot, a percentage-wise summary of the risk of bias was systematically evaluated and reported. Green, red, and yellow bars correspond to low, high, and unclear risk of bias, respectively.

The finite set of eligible studies included in this meta-analysis helped to address specific research questions related to cardiometabolic health factors associated with the subject population (see **Figure 2B**) and sedentary behaviors. There were no reports of low likelihood of bias, with all included studies having biases within at least one major domain (**Figure 4**). Three studies (16.6%) reported an unclear bias in random sequence generation representing which groups or trials the participants were allocated to, 9 studies (50%) reported allocation concealment as unclear or high-risk that specifies participants’ prior information on study design and allocation, 13 studies (72%) reported unclear or high-risk bias in the double blinding of participants and personnel contributing to difficulties in the evaluation and assessment. Blinding of outcome assessment was reported as unclear or high-risk among 10 studies (55%) that determine that the outcomes were influenced by the prior knowledge of the intervention received. 3 studies (16.6%) reported unclear or high-risk bias with incomplete outcome data, and 3 studies (16.6%) reported unclear or high-risk bias with selective reporting, which means selectively disclosing the direction of the findings to avoid false-positive outcomes. While most included studies exhibited a low risk of other biases, only 6 studies (33.3%) reported an uncertain or high risk of other biases related to research design and implementation. Several studies showed greater biases related to blinding participants and subsequent outcomes. The comprehensive evaluation of the studies included revealed that their quality was high, supporting their inclusion in the meta-analysis.

### 3.2 Yoga-related RCTs show moderate influence on biomarkers of stress-associated hypertension and disease prevention

**Figure 5:**
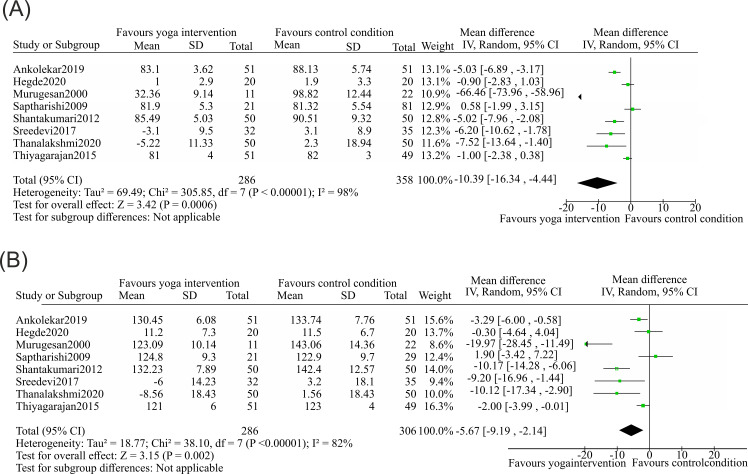
Forest plots of the effects of yoga interventions on hypertension, a factor related to a sedentary lifestyle. Data plots represented mean differences (MD) with 95% confidence intervals (CI) and were pooled using an inverse-variance random-effects model. **(A)** Diastolic blood pressure, a total of eight studies were included (yoga group *n* = 286; control group *n* = 358). A significant reduction in DBP with MD = −10.39 mmHg (95% CI −16.34 to −4.44), assessed using a two-sided Z-test, Z = 3.42, exact *p* = 0.0006, was noted, indicating outcomes favoring yoga intervention. **(B)** Systolic blood pressure included eight studies (yoga group *n* = 286; control group *n* = 306). The plot demonstrated a significant reduction in SBP favoring yoga intervention with MD = −5.67 mmHg, 95% CI −9.19 to −2.14; two-sided Z-test, Z = 3.15, *p* = 0.0020.

This meta-analysis evaluated the significant changes in blood pressure (SBP and DBP), which are the primary outcomes of interest, after yoga intervention. For our analysis, DPB data from 8 studies (64, 66, 72, 74, 75, 77–79) were selected. Among the 286 samples present in the yoga (experimental) group, and 358 that were present among the control (comparison) group, the heterogeneity [I^2^] was 98% (p <0.00001). Participants allocated in the yoga subset reported a mean decrease in blood pressure of −10.39 mmHgmmHg compared with controls, with a 95% confidence interval ranging from −16.34 to −4.44 mmHg. For SBP, the same 8 studies (64, 66, 72, 74, 75, 77–79) reported sample sizes of 286 in the intervention group and 306 in the comparison group. For SBP, the heterogeneity [I^2^] was 82% (p <0.00001). The mean difference was a −5.67 mmHg decrease (95% confidence interval −9.19--−2.14) for the intervention arm compared with the reference condition. The statistical analysis for the meta-analyses of DBP and SBP are presented as in **Figures 5A, B**.

### 3.3 Routine-based interventions impacted lifestyle modifications toward reducing type II diabetes

**Figure 6:**
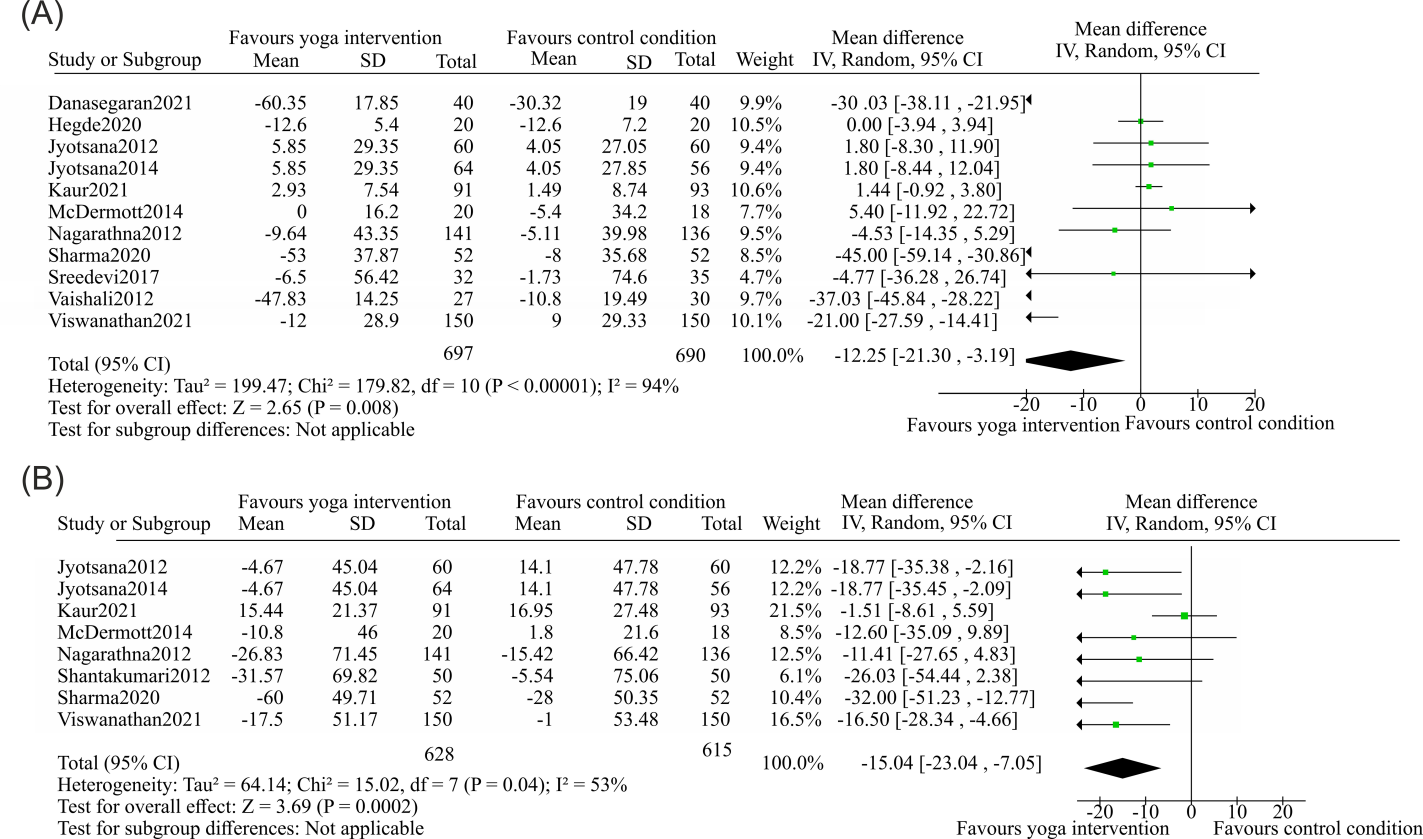
Forest plots of the effects of yoga on factors associated with type II diabetes and diseases related to a sedentary lifestyle. **(A)** Fasting blood glucose. Eleven studies were included in the meta-analysis, comprising a yoga group *n* = 697 and a control group n = 690. The plot shows a significant reduction in FBG favoring yoga intervention with MD = −12.25, 95% CI −21.30 to −3.19; two-sided Z-test, Z = 2.65, *p* = 0.008. **(B)** Postprandial blood sugar with eight included studies yoga group n = 628, and control group n = 615. The plot demonstrates a significant reduction in PPBS favoring yoga intervention with MD = −15.04, 95% CI −23.04 to −7.05; two-sided Z-test, Z = 3.69, p = 0.0002).

The analysis of 11 studies (65–70, 73, 76, 77, 80, 81) reported fasting blood glucose (FBG) as a primary outcome for the effect of yoga and its association with managing type II diabetes. The total sample comprised 697 individuals in the yoga group and 690 individuals in the control group. For FBG, the pooled WMD was −12.25 mg/dL (95% CI: −21.30 to −3.19), with I² = 94% indicating substantial heterogeneity (P < 0.00001) (**Figure 6A**).

Three studies (35, 61, 93) have reported the evaluation of postprandial blood sugar (PPBS) as a factor for monitoring blood glucose, which is also an independent factor for cardiovascular diseases. Taking this factor into consideration, the authors noted that studies focused on the PPBS as the secondary outcome measure for the study of type II diabetes. In this meta-analysis, 8 studies (67–70, 73, 75, 76, 81) reported the PPBS as a secondary outcome measure for analyzing the impact of and its association with managing type II diabetes. The PPBS analysis included 628 participants in the yoga intervention category and 615 in the control subset. In comparison with the control condition, a notable difference in the PPBS score was reported among the yoga intervention groups. The statistical difference in mean value measured for PPBS was -15.04 mg/dL (95% CI −23.04 to −7.05), and the reported I^2^ was 53% (**Figure 6B**).

12 RCTs may have used yoga intervention as an alternative medicine system that may have attenuated the impacts of T2DM. The studies revealed a decrease in FBG and PPBS levels in the yoga intervention groups, indicating the influence of yoga training as a sustainable strategy for the prophylaxis and mitigation of prediabetes and type II diabetes.

### 3.4 Daily yoga training improved cardiometabolic health

**Figure 7:**
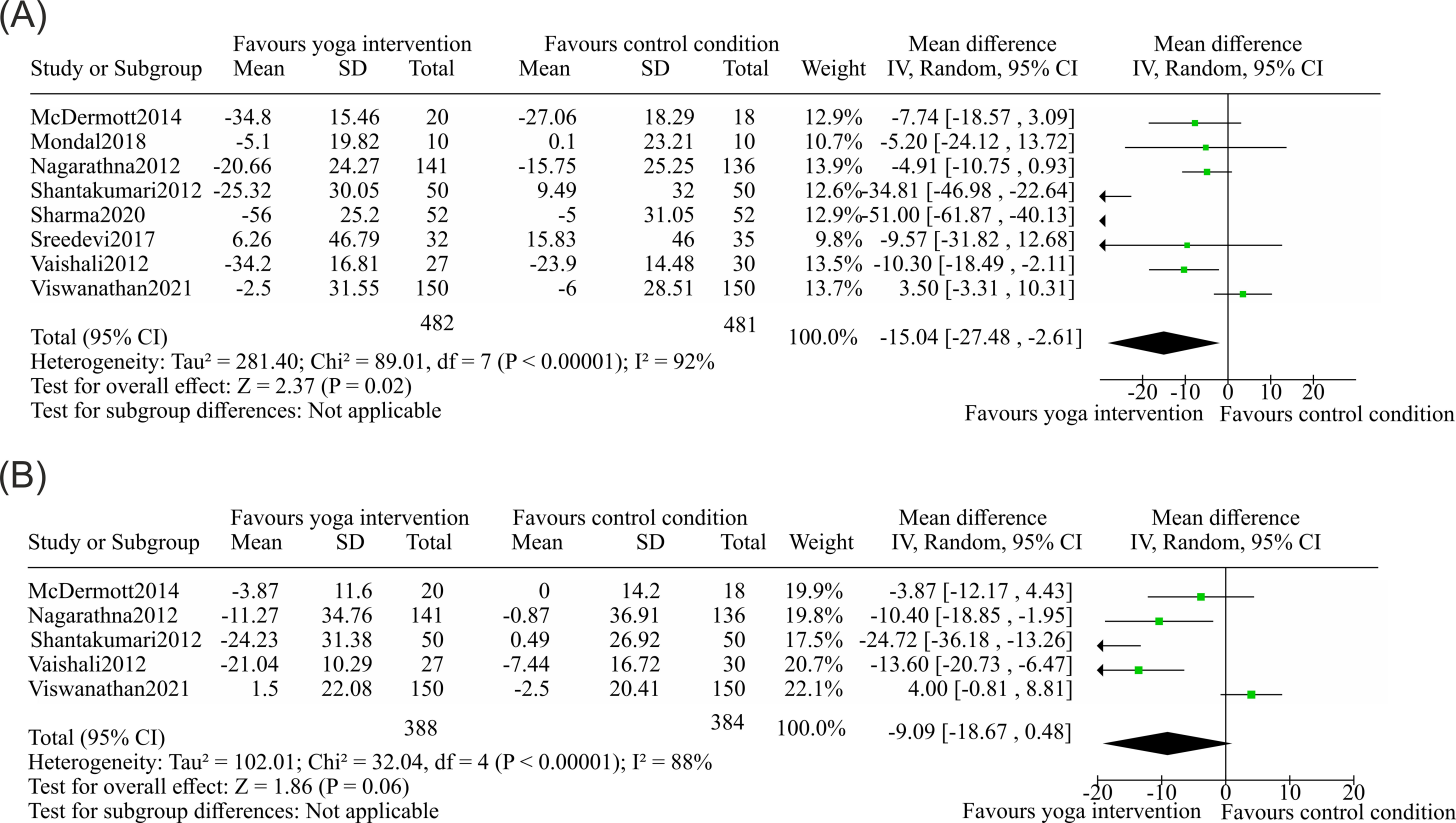
Forest plots of the effects of yoga on risk factors associated with cardiovascular diseases and diseases rooted in sedentary lifestyles. **(A)** Total cholesterol, eight studies were included for analysis comprising of yoga group n = 482 and the control group n = 481. The statistical analysis showed a significant reduction in TC favoring yoga intervention with calculated MD = −15.04, 95% CI −27.48 to −2.61; two-sided Z-test, Z = 2.37, p = 0.020). **(B)** Low-density lipoprotein. Five studies were included with yoga group *n* = 388 and control group *n* = 384. The pooled effect was not statistically significant (MD = −9.09, 95% CI −18.67 to 0.48; two-sided Z-test, Z = 1.86, *p* = 0.060). The MD is −9.09, 95% CI −18.67 to 0.48; two-sided Z-test, Z = 1.86, p = 0.060, which was not statistically significant.

In this meta-analysis, 8 studies (70, 71, 73, 75–77, 80, 81) reported the measurement of total cholesterol as a primary outcome for the role of yoga interventions in modulating cardiovascular risk factors. The sample sizes of the included studies were 482 volunteering individuals in the yoga group and 481 individuals in the control group. The pooled weighted mean difference (WMD) was −15.04 mg/dL (95% CI −27.48--−2.61; P for heterogeneity <0.00001 and I^2^ = 92%) (**Figure 7A**). Five studies (70, 73, 75, 80, 81) reported LDL levels as a factor for cardiovascular risk factors. The sample sizes of the included studies were 388 subjects in the yoga group and 384 subjects in the control group. The pooled weighted mean difference (WMD) was −9.1 mg/dL (95% CI −18.68 to 0.48; P for heterogeneity <0.00001 and I^2^ = 88%) (**Figure 7B**).

Elevated levels of serum total cholesterol (TC) is considered the primary contributor of developing coronary atherosclerosis, while low-density lipoprotein (LDL) has also been considered the main risk factor for CVD, as it plays a major role in atherosclerosis pathogenesis (94).The 5 RCTs on LDL had a P value of 0.06, suggesting consistent yoga intervention strategies; nevertheless, for robust evaluation, a broader sample size is needed to obtain conclusive results with a pooled combined effect size.

### 3.5 Methodological sources of heterogeneity across studies

From the overall analysis across the included randomized controlled trials, consistent improvements in cardiometabolic outcomes, particularly lowering the systolic and diastolic blood pressure, fasting and postprandial blood glucose, and lipid profile were observed. Although the direction of effect consistently favored yoga, the detailed GRADE assessment showed that the overall certainty of evidence is limited (**Table 3**).

**Table 3:** Pooled effects of meta-analysis and GRADE evidence certainty for yoga interventions on cardiometabolic health.

Reported Outcome Measures	Mean difference Pooled effect from meta-analysis	Result implications	Number of studies included	Number of participants	Certainty of Evidence (GRADE)
Diastolic Blood Pressure	−10.39 mmHgmmHg	Favors yoga	8	644	Very Low
Systolic Blood Pressure	−5.67 mmHg	Favors yoga	8	592	Low
Fasting Blood Glucose	−12.25 mg/dL	Favors yoga	11	1387	Very Low
Postprandial Blood Glucose	-15.04 mg/dL	Favors yoga	8	1243	Low
Total Cholesterol	−15.04 mg/dL	Favors yoga	8	963	Very Low
Low-density lipoprotein	−9.1 mg/dL	Favors yoga	5	772	Very Low

The certainty of evidence in the analyses ranged from low to very low due to methodological limitations, substantial heterogeneity (I² ranging from 53% to 98%), and imprecision of pooled estimates. The primary reasons analyzed showed high heterogeneity, suggesting considerable variation in yoga styles, session durations, comparator groups, and participant characteristics, and persistent risks of bias related to inadequate randomization and lack of blinding. Across the included studies, many studies did not specify the type of yoga employed for the population under study, and substantial heterogeneity was observed in duration of yoga practices, ranging from days to 9 months, frequency of sessions, weekly once to five times per week, and length of yoga sessions from 30minutes to 90 minutes. In the included studies, a substantial proportion of interventions were either multi-component, including asanas, pranayama, meditation practices, and relaxation, asana-dominant interventions or inadequately described that may be considered a factor to methodological heterogeneity. Subgroup analysis for the type of yoga intervention and the duration of intervention for each outcome of sedentary life associated with cardiometabolic disorders were further evaluated using the same random-effects model.

**Figure 8:**
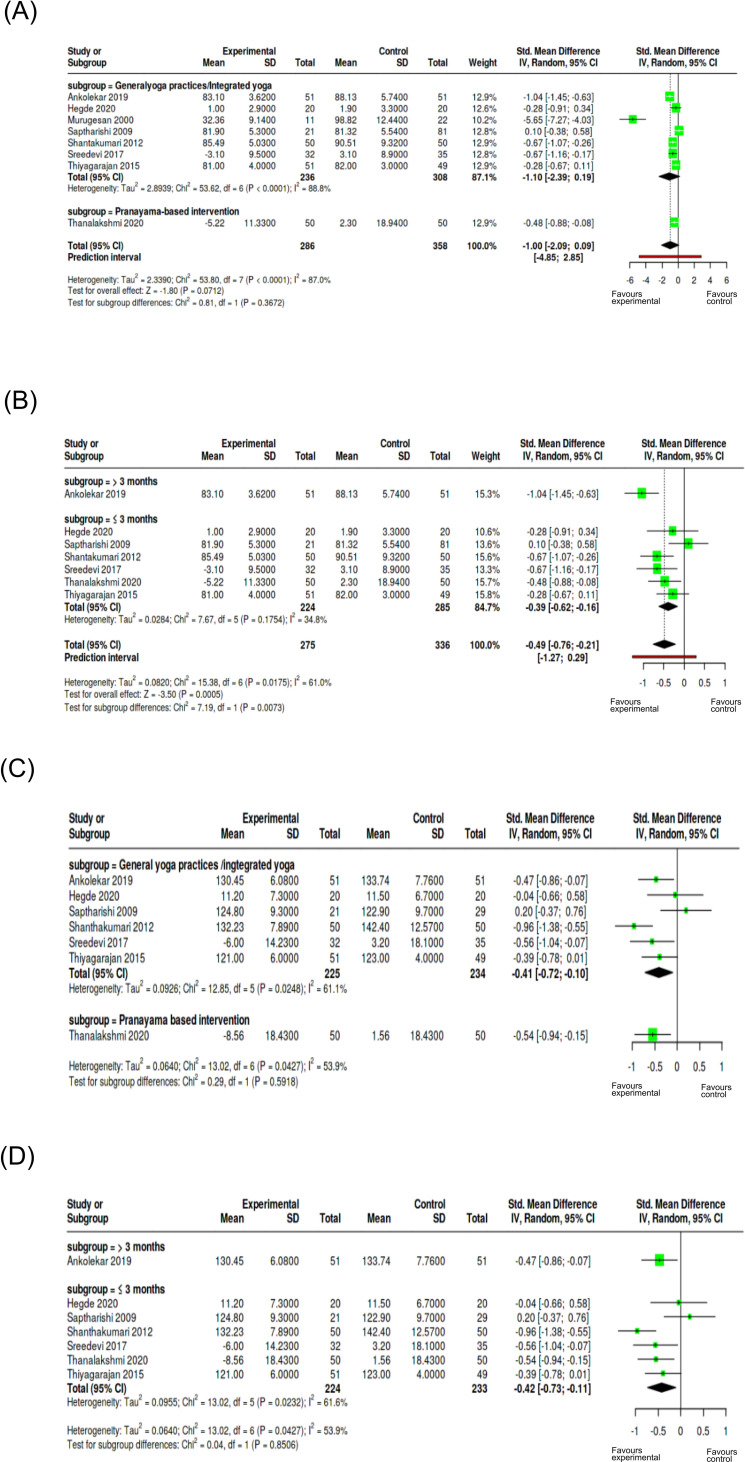
Subgroup analysis of blood pressure outcomes. **(A)** DBP based on intervention type; **(B)** DBP based on duration; **(C)** SBP based on intervention type; **(D)** SBP based on duration. Subgroups were defined by general/integrated yoga vs pranayama-based interventions and duration (≤3 months vs >3 months. Effect sizes are expressed as standardized mean differences (SMD) with 95% confidence intervals using a random-effects model.

**Figure 9:**
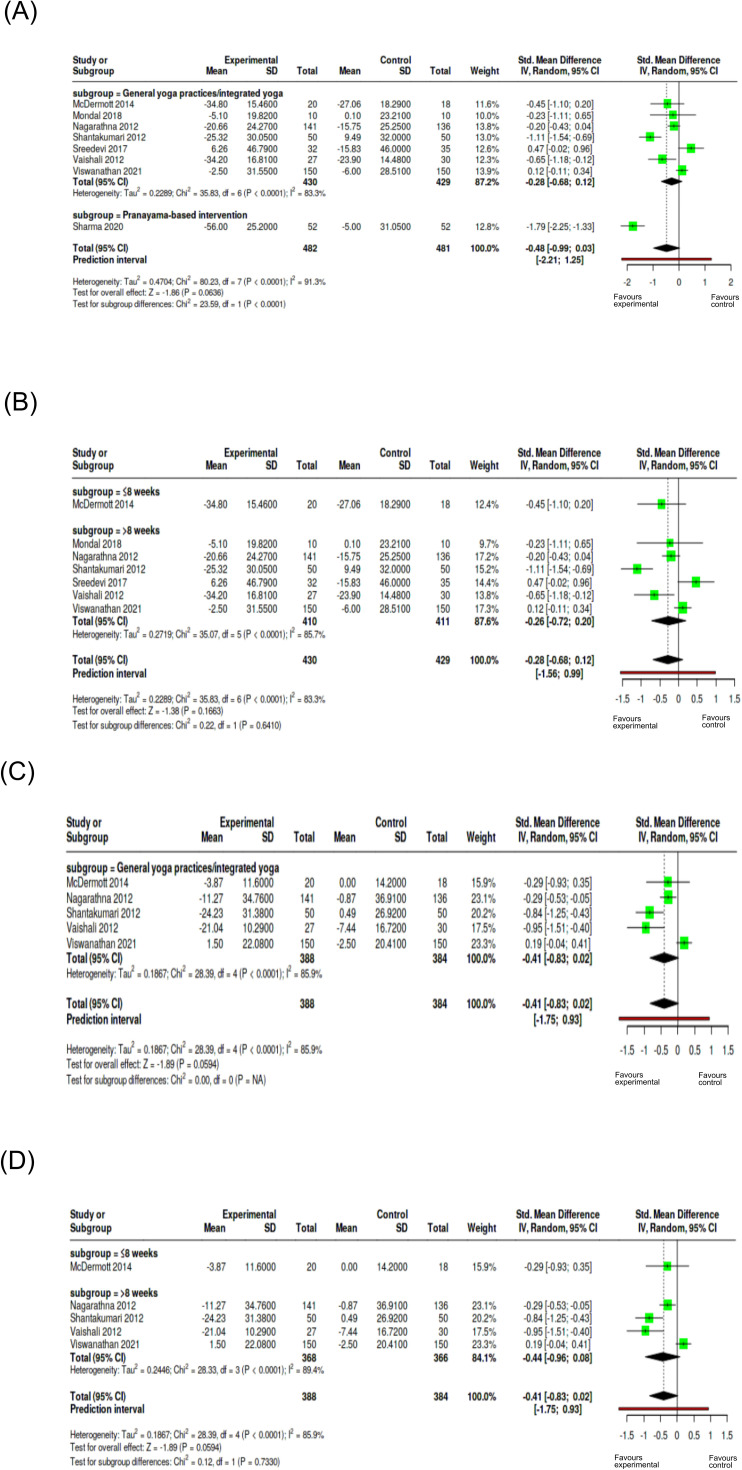
Subgroup analysis of glycemic outcome measures. **(A)** Effect on FBG based on intervention type; **(B)** Effect on FBG based on duration of intervention; **(C)** PPBS based on yoga intervention; **(D)** PPBS based on duration. Subgroups were defined general/integrated yoga vs pranayama-based interventions and duration (≤8 weeks vs >8 weeks). Effect sizes are expressed as standardized mean differences (SMD) with 95% confidence intervals using a random-effects model.

**Figure 10:**
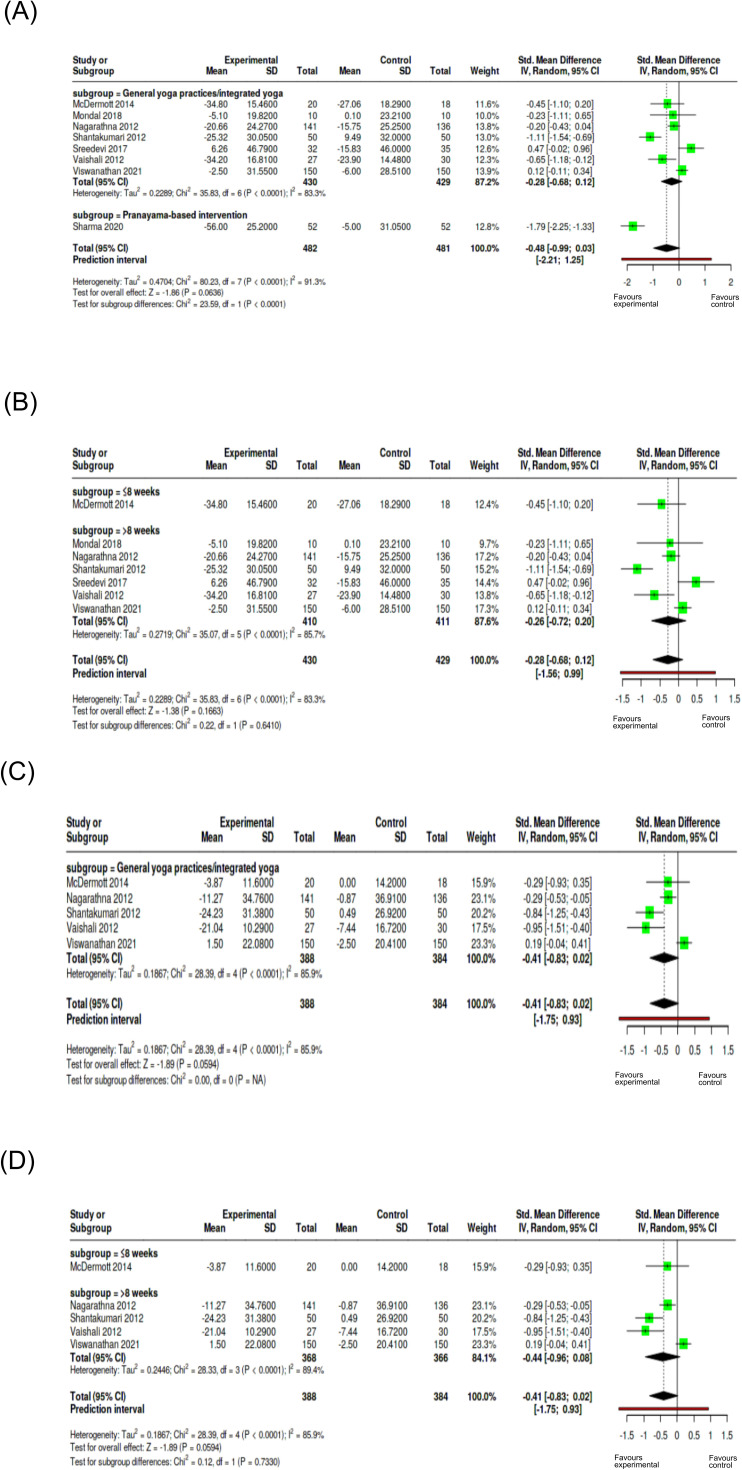
Subgroup analysis of lipid profiles as cardiometabolic outcomes. **(A)** TC based on intervention type; **(B)** TC based on duration; **(C)** LDL based on intervention type; **(D)** LDL based on duration Subgroups were defined as general/integrated yoga vs pranayama-based interventions and duration (≤8 weeks vs >8 weeks). Effect sizes are expressed as SMD with 95% confidence intervals using a random-effects model.

The subgroup analysis based on yoga intervention on DBP showed a reduction of DBP on yoga group across both intervention categories. The statistical analysis of test for subgroup differences was not significant (Chi² = 0.81, p = 0.367), indicating the type of yoga intervention has no significant influence on DBP measures (**Figure 8A**). The duration-based analysis (Chi² = 7.19, p = 0.0073) indicated that longer duration of interventions (> 3 months) significantly reduced DBP (**Figure 8B**). Even though for SBP, both the intervention type demonstrated significant reductions in SBP values, test for subgroup differences (Chi² = 0.29, p = 0.592) indicated the type of intervention did not modify SBP effects (**Figure 8C**). Similarly, duration-based intervention showed reduction in SBP in both the categories but the test for subgroup analysis was not statistically significant (Chi² = 0.04, p = 0.8506), that is, intervention duration did not affect SBP outcome measures (**Figure 8D**). The subgroup analysis on yoga intervention for FBG showed no significant difference between general yoga practices or integrated yoga and pranayama-based interventions (**Figure 9A**). The subgroup analysis on duration showed a significant subgroup difference with yoga interventions of more than 8 weeks contributing to a reduction in FBG compared to short-duration interventions (**Figure 9B**). Considering PPBS, both the pranayama-based interventions and general or integrated yoga interventions showed statistically significant reduction. Heterogeneity was 4.6%, indicating consistency across the studies. The test for subgroup differences showed that type of yoga intervention has no significant influence on PPBS (**Figure 9C**). Results also showed that the duration of yoga intervention does not significantly contribute to PPBS reduction (**Figure 9D**). For TC, the single pranayama-based intervention suggested a marked reduction in the yoga group. Test for subgroup differences was statistically significant (Chi² = 23.59, p < 0.0001), suggesting that the role of type of yoga interventions in managing the level of total cholesterol (**Figure 10A**). However, the duration-based analysis on TC was not statistically significant with respect to intervention duration (**Figure 10B**). For LDL, intervention type included only general yoga practices/integrated yoga studies, and subgroup testing based on intervention was not possible (**Figure 10C**). The test for subgroup differences showed that the duration of intervention did not significantly influence outcomes for LDL (**Figure 10D**). Sensitivity analyses showed the robust effects across outcomes, with duration influencing DBP and FBG. The interventions lasting >3 months show greater reductions in DBP while shorter-duration interventions (≤8 weeks) demonstrate greater reductions in FBG levels. No significant differences were observed in other outcome measures in sensitivity analysis (**Supplementary Figures 1–3**). Moreover, control conditions reported were mostly usual care or no treatment, while fewer used standard medication or active exercise as comparators and passive control groups that made difficult to classify for subgroup analysis. As these studies compare yoga interventions and their effects with less intensive controls, the pooled estimate favored yoga interventions when compared with more active treatments like exercise, contributing to heterogeneity in result interpretations. An exploratory subgroup analysis on type of comparison group and frequency of intervention was performed to examine the sources of heterogeneity (**Supplementary Table 2**).

## 4 Discussion

This study aimed to evaluate how yoga interventions may influence sedentary lifestyle-associated cardiometabolic diseases and to explore their possible contribution to sustainable health goals. The 18 included studies showed substantial heterogeneity in yoga interventions, due to reported multicomponent interventions including asanas, pranayama, meditation practices, and relaxation, asana dominant interventions that focus on postures with limited breathing exercise, pranayama only interventions and specific protocols such as Sudarshan Kriya. The session durations reported vary from 30–90 minutes, frequency varies from 2 to 7 days/week, and total length varies from 8 weeks to 9 months. This variability likely explains the inconsistent effect sizes related to outcomes reported in this study, even though the multicomponent programs showed broader associations, while pranayama-only or short intensive protocols appeared limited mainly to autonomic changes. Considering these substantial methodological heterogeneity factors, yoga intervention components, duration, frequency, participant characteristics, and comparator arms, the meta-analysis was conducted using a pre-specified random-effects model, as the purpose of the meta-analysis is to provide generalizations of outcomes on yoga interventions in non-communicable diseases across a broad population.

The meta-analysis reported that yoga training has been associated with reductions in multiple factors associated with sedentary lifestyle practices, such as T2DM management and prevention, hypertension, and biomarkers of cardiovascular diseases, including lipid profiles, blood pressure, and glucose levels. However, the certainty of the evidence was rated as low to very low according to the GRADE assessment, which is largely attributable to high heterogeneity across studies and variability in yoga protocols, participant characteristics, and intervention duration. The risk-of-bias assessment revealed the highest concerns in the blinding of participants and personnel (performance bias), which is a common challenge in yoga trials, as the nature of the intervention makes blinding difficult and may influence participants’ expectations or behaviors under conditions such as test versus control or placebo. Systematic differences in age between groups in the selected studies may have further affected the accuracy of the estimated intervention effects. Though statistically significant results favoring yoga interventions were observed across the subset of meta-analysis studies, their association to translate into clinically meaningful improvements in type 2 diabetes management or prevention, hypertension control, or cardiovascular disease risk reduction remains unclear for broad improvements in quality of life or therapeutic benefits across large populations, particularly in developing nations such as India. A consistent observation across the reviewed studies is the need for well-designed protocols that specify the type, frequency, and duration of yoga asanas to serve yoga practices as a complementary approach alongside conventional medical treatments, consistent with the principles of personalized medicine.

This meta-analysis observed that yoga training was associated with a statistically significant reduction in systolic and diastolic blood pressure. The magnitude of any effect appeared to vary depending on the style of hatha yoga, frequency, and duration of practice. As the pathophysiology of hypertension involves multiple interrelated mechanisms, these findings suggest a possible modest benefit and were linked to a meaningful reduction in cardiovascular disease risk in sedentary populations. This indicates yoga may be a choice as an adjunct intervention alongside standard antihypertensive medication, with improved physical fitness, reducing mortality and morbidity with lifestyle modifications, supporting improved physical fitness. Although the current evidence did not consider yoga practices as alternative to pharmacological treatments, yoga-based community programs were suggested as a sustainable solution to reduce the burden of hypertension in sedentary populations due to its role in improving physical activity. Similarly, the included studies indicated the role of routine yoga practice with modest improvements in glucose regulation, with a greater consistency observed for postprandial blood sugar (PPBS) than fasting blood glucose (FBG). The low heterogeneity score for the PPBS is related to consistency in attributes across the selected studies for this outcome measurement. Even though yoga practices reduce the risk of developing type 2 diabetes, the current evidence does not provide detailed insights into how regular yoga practice can improve insulin sensitivity and restore pancreatic beta-cell function. Few studies reported HbA1c as an outcome measure and given the variations in yoga intervention duration and protocols, this review did not focus on HbA1c levels. The present study also evaluated the potential role of yoga interventions in addressing cardiovascular disease (CVD)-associated risk factors. Increases in blood cholesterol levels have been implicated as a leading cause of the elevated risk of CVD. The observed effect across the included studies suggested that yoga training was associated with modest reductions in cholesterol levels; the low certainty of evidence is due to factors such as the style of yoga and the characteristics of the intervention. More consistent, high-quality studies with clearly defined optimal intervention parameters are recommended to clinically determine the impact of yoga training on cholesterol reduction.

To address the observed heterogeneity across the outcomes reported in this study, subgroup and sensitivity analyses based on intervention characteristics and duration were performed to identify sources of study variability factors and to evaluate the robustness in the pooled estimates. Exploratory analysis on comparison group, and frequency of intervention and population-related variables were also undertaken in this study. The effect of yoga was reportedly less influenced by the nature of the study. Sensitivity analysis of DBP and FBG showed duration-dependent variability; longer duration for reduction in DBP, while shorter duration influences FBG outcome. These factors can be considered clinically important for hypertension management and glucose reduction in diabetic conditions. The analysis indicated larger effect sizes with usual care or minimal intervention than with active comparison groups, suggesting the influence of daily life activities. For outcomes that showed heterogeneity even after the subgroup or sensitivity analysis, need for attention in evaluation, and acknowledged that the effects indicated trends of yoga practices for different outcomes rather than precise effect estimates and the magnitude and consistency may vary depending on the protocol design for yoga intervention studies. An indicative association of yoga interventions may have roles in improving cardiometabolic disorders-related health rather than focusing on the treatment effects.

The preliminary analysis suggested that yoga may serve as an adjunct approach rather than a primary strategy for managing cardiometabolic risk factors in sedentary or healthy populations. The effect size variability sizes observed across clinical outcomes underscores the need for standardized yoga intervention protocols and highlights the importance of further research to evaluate long-term effects. The findings of this review are consistent with those of previous meta-analytic evidence and review-based studies that assessed the outcome-based analysis of yoga interventions on reducing the adverse factors associated with cardiometabolic disorders. (95, 96). Yoga practice has been hypothesized to influence autonomic nervous system function by reducing sympathetic tone and increasing parasympathetic activity, which could theoretically attenuate certain molecular or genetic biomarkers associated with cardiovascular and coronary artery disease (97). Such evidence was not directly reported in this analysis. The present meta-analysis was limited to studies published up to 2024 that met the pre-specified inclusion criteria. Many of the included reports had important methodological limitations, including a lack of robust randomization in some trials, inherent difficulties with blinding of participants and personnel (due to the nature of yoga interventions), and relatively short follow-up durations. These factors remain as a challenge in yoga research and likely contributed to the high risk of performance bias and the overall low to very low certainty of evidence according to the GRADE assessment. A formal assessment of publication bias including funnel plot asymmetry or Egger’s regression test, was not performed due to the limited number of studies in the outcome assessment categories. This may influence the pooled estimates and chances of overestimating the observed beneficial trends of yoga interventions. Restricting the study to Indian populations allowed synthesis of evidence to align with the study’s aim of incorporating sustainable public-health strategies in developing nations. Several RCTs were designed to assess the safety considerations, feasibility, and therapeutic implications of yoga asanas and breathing practices on chronic coronavirus infection recovery (98) and in other clinical conditions to cultivate prosociality feelings and behaviors in addition to reports that implicated the limited prosocial effects of mindfulness practices and associated societal impacts (99). Results highlight the need for prioritizing methodological rigor, standardized protocols, other key improvements on stronger randomization procedures, the use of active comparators to address blinding challenges, standardized and clearly described protocols, and longer follow-up periods to reduce heterogeneity, enhance precision, and reduce inconsistency in future yoga intervention studies on cardiometabolic outcome measures. In a broader sense, the incorporation of yoga training in public health initiatives and clinical interventions can lower the risk of mortality and morbidity connected with sedentary lifestyles, the available certainty of evidence using GRADE analysis was not satisfactory for determining the long-term impact of yoga training in public health domains. Owing to considerable heterogeneity in the outcome measures reported in this study, it may therefore be recommended to include a larger population and more rigorous studies to confirm and evaluate the prolonged impact of yoga training on sedentary lifestyle-associated factors and diseases. Further investigations are needed to determine the choice of yoga asanas, session duration, and standardized trials that should be employed for specific disorders and to elucidate the therapeutic effects of yoga interventions across different populations.

Diverse studies suggest the inclusion of regular yoga into conventional healthcare and rehabilitation programs, as it is regarded as Traditional, Complementary, and Alternative Medicine (TCAM)-based mind-body medicine provided with holistic and preventive healthcare approaches, especially in sports rehabilitation, immune modulation, and disease prevention to an extent. Although yoga has a long-term impact on several aspects of mind and body, the clinically relevant outcomes perspectives across diverse populations need to be estimated. The outcomes reported in this study highlighted the need for novel approaches for integrating yoga into government health initiatives prioritizing yoga training as a public health strategy plan. Being a technique rooted in cultural heritage, the scientific validation of different aspects of yoga training would bridge the gap between lifestyle management and clinical aspects, tailoring them to diverse demographics and cultural backgrounds.

The number of yoga schools, training centers, and therapeutic programs have increased significantly, leading to a potential wider implementation of yoga-based interventions for health and wellness management. This growing expansion further highlights the need for rigorous studies focusing on cardiometabolic diseases such as diabetes within zones and countries with large populations. From the outcome-based analysis presented in this study, we intend to explore and standardize yoga-based therapeutic intervention protocols as sustainable approaches for improving specific health and well-being of student and working populations. By synthesizing evidence from the meta-analysis study focused on studies, a next step is to develop structured intervention frameworks for research integrating specific yogic practices such as Swastikasana, Vajrasana, Shavasana (100, 101), sun salutations (102) and Trataka practices (manuscript in preparation) for health and well-being and contributing to the UN Sustainable Development Goal (SDG) 3.

## 5 Limitations of the study

This meta-analysis has several important limitations that should be considered when interpreting the outcomes. Firstly, the pre-registration of the trial protocol with the established reporting guidelines such as CONSORT further strengthens the credibility of the findings and reduces the risk of reporting bias. Secondly, the literature search was restricted to published peer-reviewed articles; some potentially relevant studies may have been missed even though some potentially relevant studies may have been missed, even with consistent inclusion criteria. Third, the methodological quality of the included RCTs was generally low. The highest risk of bias was observed in the blinding of participants and personnel (performance bias), with additional concerns regarding randomization and allocation concealment in several trials that are inherent to behavioral interventions such as yoga leading to low certainty of evidence. The statistical heterogeneity across reported outcomes (was high due to variability in yoga intervention protocols, frequency, and duration even after subgroup and sensitivity analysis. Publication bias was not formally assessed due to the limited number (fewer than 10) of included studies in the pooled outcome estimates, limiting the reliability of funnel plots and statistical tests such as Egger’s regression. Lastly, all the 18 included studies were conducted exclusively in India. The pooled estimates derived from this meta-analysis restrict the generalizability of the findings as they cannot be related to populations in other countries, ethnicities, and healthcare systems, as the yoga practices, daily life activities, and cardiometabolic risk profiles vary accordingly. As India reported a high burden of sedentary lifestyle-related non-communicable diseases in recent years and as one of the leading contributors to research in yoga-based interventions, the study’s intention is a consistent evaluation of different yoga-based interventions, following traditional practice frameworks, thereby providing a relevant evidence base for the ongoing research design.

## 6 Conclusions and future directions

In summary, this review indicated yoga-based interventions have been associated with favorable trends across several cardiometabolic health markers linked to sedentary lifestyles associated with health complications. The exploration may suggest that some yoga protocols may serve as an integrative, non-pharmacological approach to conventional treatments for conditions such as hypertension, cardiovascular disease, and type 2 diabetes mellitus in sedentary populations. However, the evidence reported in this study may not be adequate to draw broad conclusions as it may affect the precision of the pooled estimates. To assess the robustness of the findings, and to further explore heterogeneity sources subgroup and sensitivity analyses were performed. The results reported were considered as indicative trends of yoga interventions in cardiometabolic disorders rather than definitive conclusions regarding the overall effectiveness of yoga interventions.

Due to this, standardized and reproducible descriptions of interventions, such as the Template for Intervention Description and Replication [TIDieR] checklist) need to be considered before analysis (103).

Future studies should highlight research based on high-quality RCTs, with broader sample size, to evaluate the substantial influence of regular yoga and meditation practices into daily activities and in clinical cases to study their impact on improving motor coordination in Parkinson’s disease and Alzheimer’s disease patients. Additional research studies can evaluate how yoga training influences lifestyle modifications during COVID-19 outbreaks and on lockdown days to promote good health and well-being and extend these findings to elaborate on the influence of yoga training in regulating stress to cope with work-life balance. Further studies employing neuroimaging techniques would be valuable to examine the cognitive and neural effects of yoga and meditation and to study neural correlates associated with yoga training among short-term versus long-term practitioners and male versus female practitioners. Standardization of protocols and intervention criteria should be the next target for comparative analysis on the sustained effects of yoga training across heterogeneous multi country populations without any adverse effects to enhance the validity of the findings.

## Data Availability

The original contributions presented in the study are included in the article/**Supplementary Material**. Further inquiries can be directed to the corresponding author.
