# Supplementary material for: Yoga interventions and randomized controlled trials: key issues and the centered impact on sedentary lifestyle-associated cardiometabolic disorders
Source: Front Clin Diabetes Healthc. 2026 Apr 24;7:1805160. doi: 10.3389/fcdhc.2026.1805160 (PMC13152826; doi:10.3389/fcdhc.2026.1805160)
Supplement: Supplementary file 1 [file DataSheet1.docx]

Supplementary Material for

**Yoga interventions and randomized controlled trials: key issues and the centered impact on sedentary lifestyle-associated cardiometabolic disorders**

**Rakhi Radhamani^1^, Shyam Diwakar^1,2*^**

^1^ Amrita Mind Brain Center, Amrita Vishwa Vidyapeetham, Amritapuri Campus, Kollam, Kerala, India

^2^ Department of Electronics and Communication Engineering, Amrita Vishwa Vidyapeetham, Amritapuri Campus, Kollam, Kerala, India

*** Correspondence:**Shyam Diwakar
[shyam@amrita.edu](mailto:shyam@amrita.edu)

Supplementary Table 2. Exploratory Subgroup analysis of the included studies for sources of heterogeneity assessment

| Subgroup | Study characteristics | Observation across the outcome measures | Interpretation |
| --- | --- | --- | --- |
| Type of comparator | Usual care  Minimal intervention  Exercises  Medication | When yoga was compared with usual care, larger effect sizes were observed | The pooled effect can be due to the activities of daily life apart from yoga interventions. |
| Intervention frequency | Varies in all studies, typically less than or equal to 3 sessions or greater than equal to 5 sessions in a week | High frequency contributed to positive outcomes in yoga interventions | Frequency-dependent effect on outcome measures noted |
| Type of population | Varies according to outcome assessment. Type 2 diabetes, Hypertension, Cardiometabolic disease populations | Diastolic Blood Pressure changes in hypertension focused studies | Indication of disease severity may affect overall effects of yoga intervention |


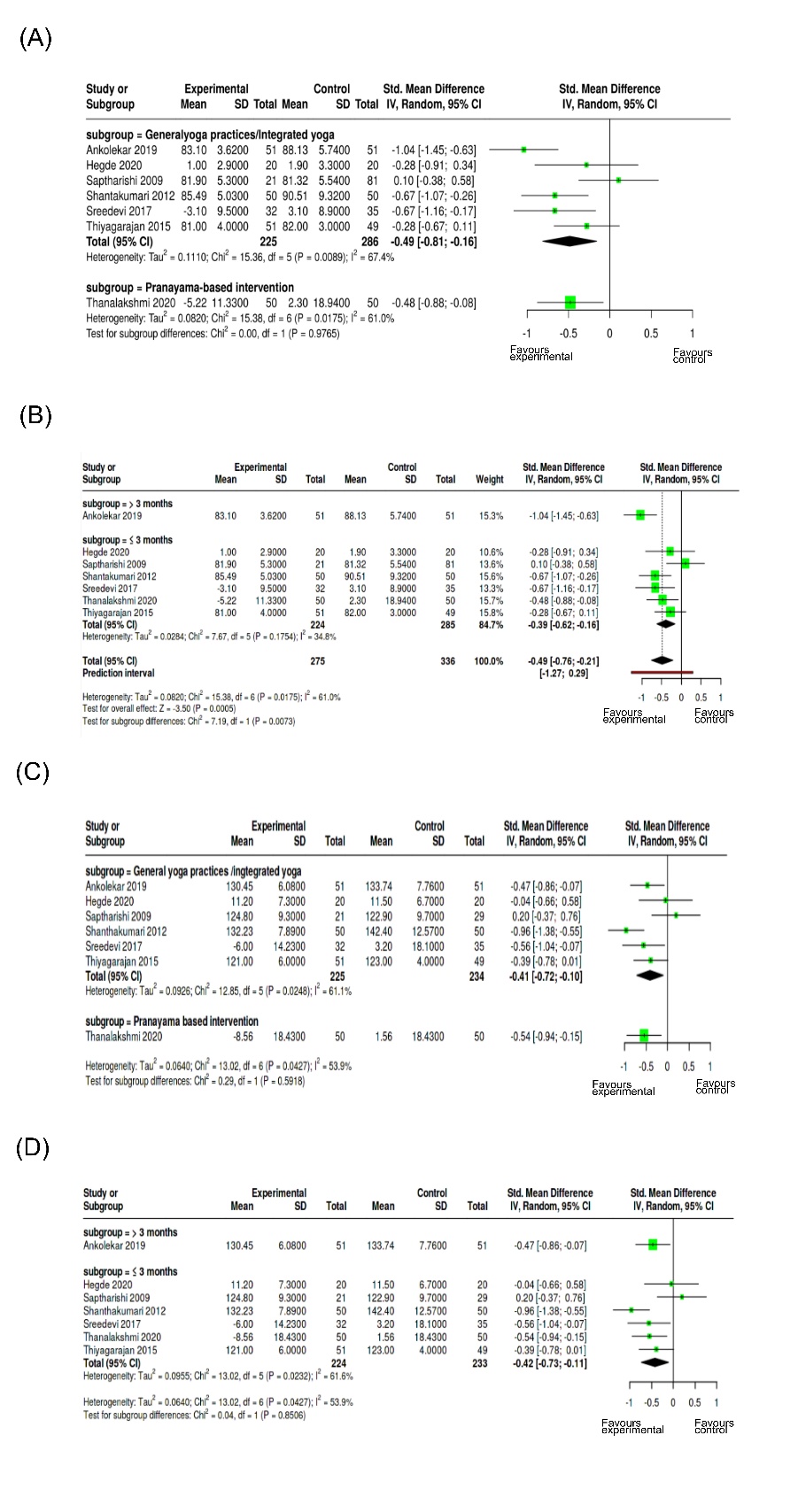


Supplementary Figure 1: Sensitivity analysis of blood pressure outcomes. A) DBP based on intervention type; (B) DBP based on duration; (C) SBP based on intervention type; (D) SBP based on duration.


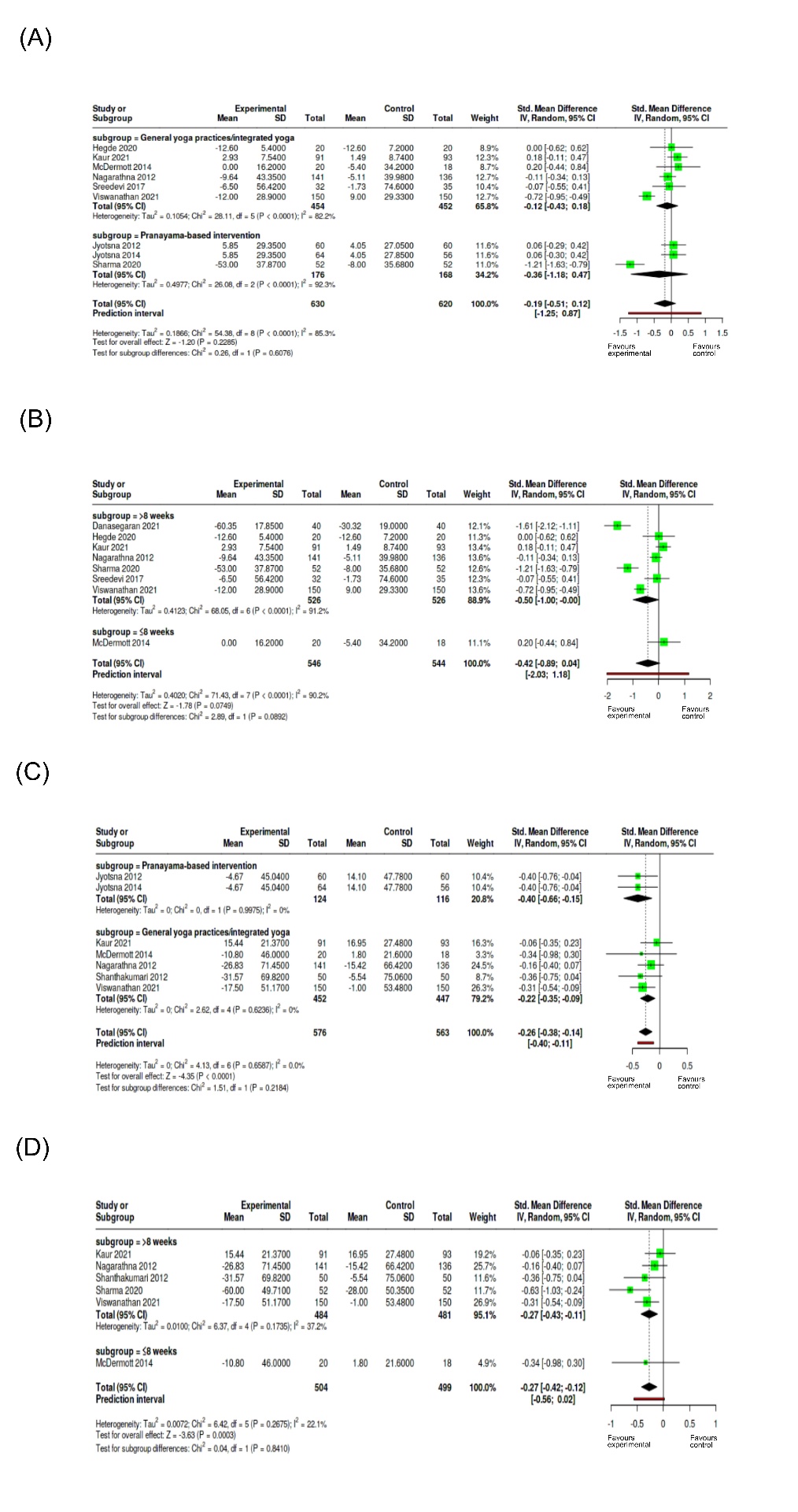


Supplementary Figure 2: Sensitivity analysis of glycemic outcome measures. A) Effect on FBG based on intervention type; (B) Effect on FBG based on duration of intervention; (C) PPBS based on yoga intervention; (D) PPBS based on duration.


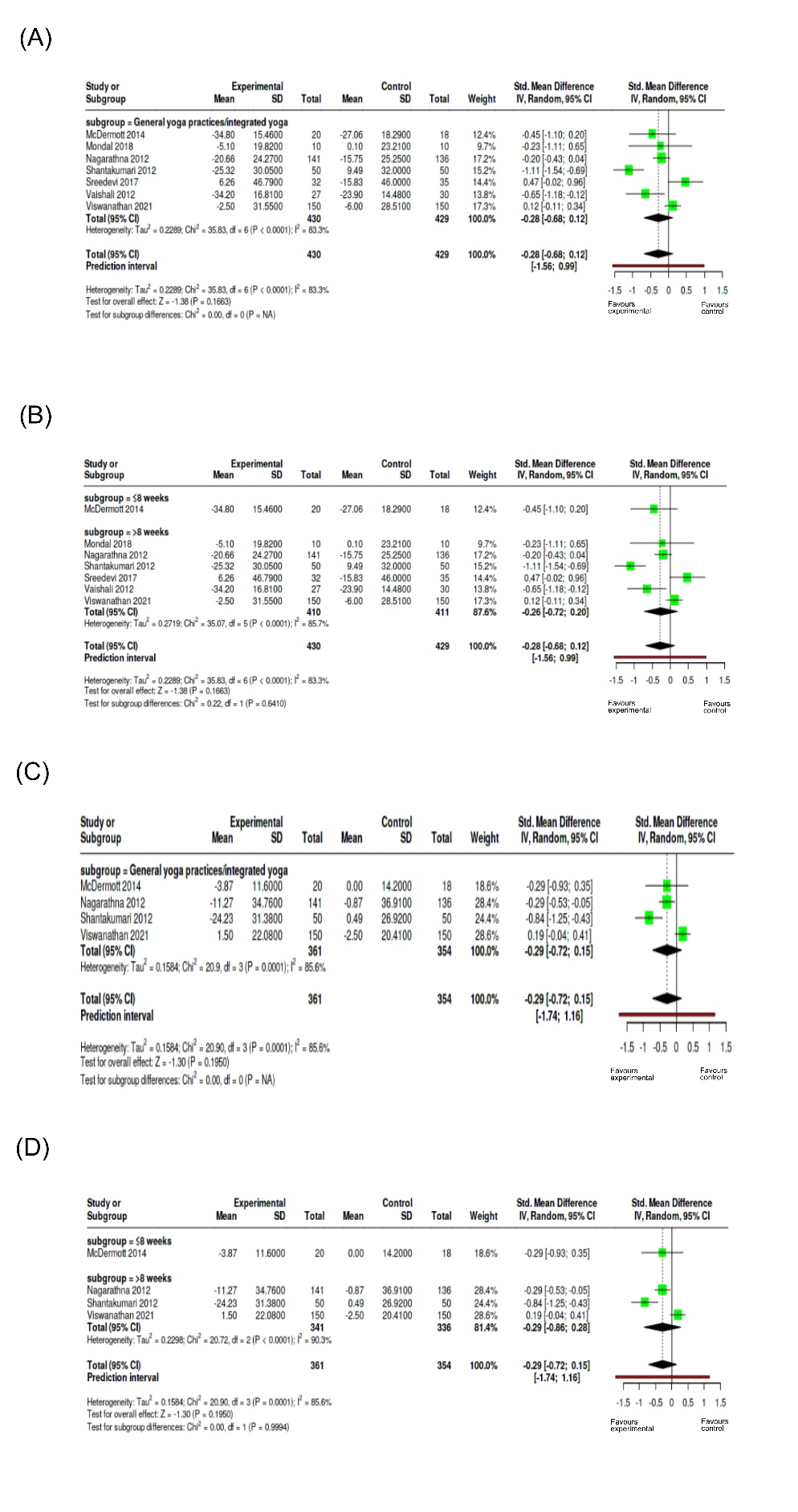


Supplementary Figure 3. Sensitivity analysis of lipid profiles as cardiometabolic outcomes. (A) TC based on intervention type; (B) TC based on duration; (C) LDL based on intervention type; (D) LDL based on duration.


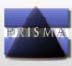
**PRISMA 2020 Checklist**

Supplementary Table 1: PRISMA Checklist

| **Section and Topic** | **Item #** | **Checklist item** | **Location where item is reported (page number)** |
| --- | --- | --- | --- |
| **TITLE** | | |  |
| Title | 1 | Identify the report as a systematic review. | Page 1 |
| **ABSTRACT** | | |  |
| Abstract | 2 | See the PRISMA 2020 for Abstracts checklist. | Page 1 |
| **INTRODUCTION** | | |  |
| Rationale | 3 | Describe the rationale for the review in the context of existing knowledge. | Page 2-3 |
| Objectives | 4 | Provide an explicit statement of the objective(s) or question(s) the review addresses. | Page 3 |
| **METHODS** | | |  |
| Eligibility criteria | 5 | Specify the inclusion and exclusion criteria for the review and how studies were grouped for the syntheses. | Page 4 |
| Information sources | 6 | Specify all databases, registers, websites, organisations, reference lists and other sources searched or consulted to identify studies. Specify the date when each source was last searched or consulted. | Page 4 |
| Search strategy | 7 | Present the full search strategies for all databases, registers and websites, including any filters and limits used. | Page 4 |
| Selection process | 8 | Specify the methods used to decide whether a study met the inclusion criteria of the review, including how many reviewers screened each record and each report retrieved, whether they worked independently, and if applicable, details of automation tools used in the process. | Page 4 |
| Data collection process | 9 | Specify the methods used to collect data from reports, including how many reviewers collected data from each report, whether they worked independently, any processes for obtaining or confirming data from study investigators, and if applicable, details of automation tools used in the process. | Page 4,5,6 |
| Data items | 10a | List and define all outcomes for which data were sought. Specify whether all results that were compatible with each outcome domain in each study were sought (e.g. for all measures, time points, analyses), and if not, the methods used to decide which results to collect. | Page 4,5,6 |
|  | 10b | List and define all other variables for which data were sought (e.g. participant and intervention characteristics, funding sources). Describe any assumptions made about any missing or unclear information. | Page 4 |
| Study risk of bias assessment | 11 | Specify the methods used to assess risk of bias in the included studies, including details of the tool(s) used, how many reviewers assessed each study and whether they worked independently, and if applicable, details of automation tools used in the process. | Page 6 |
| Effect measures | 12 | Specify for each outcome the effect measure(s) (e.g. risk ratio, mean difference) used in the synthesis or presentation of results. | Page 4,5,6 |
| Synthesis methods | 13a | Describe the processes used to decide which studies were eligible for each synthesis (e.g. tabulating the study intervention characteristics and comparing against the planned groups for each synthesis (item #5)). | Page 4,5,6 |
|  | 13b | Describe any methods required to prepare the data for presentation or synthesis, such as handling of missing summary statistics, or data conversions. | n.a |
|  | 13c | Describe any methods used to tabulate or visually display results of individual studies and syntheses. | Page 6 |
|  | 13d | Describe any methods used to synthesize results and provide a rationale for the choice(s). If meta-analysis was performed, describe the model(s), method(s) to identify the presence and extent of statistical heterogeneity, and software package(s) used. | Page 4,5,6 |
|  | 13e | Describe any methods used to explore possible causes of heterogeneity among study results (e.g. subgroup analysis, meta-regression). | Page 6 |
|  | 13f | Describe any sensitivity analyses conducted to assess robustness of the synthesized results. | Page 6 |
| Reporting bias assessment | 14 | Describe any methods used to assess risk of bias due to missing results in a synthesis (arising from reporting biases). | Page 6 |
| Certainty assessment | 15 | Describe any methods used to assess certainty (or confidence) in the body of evidence for an outcome. | Page 6 |
| **RESULTS** | | |  |
| Study selection | 16a | Describe the results of the search and selection process, from the number of records identified in the search to the number of studies included in the review, ideally using a flow diagram. | Page 8 |
|  | 16b | Cite studies that might appear to meet the inclusion criteria, but which were excluded, and explain why they were excluded. | Page 4 |
| Study characteristics | 17 | Cite each included study and present its characteristics. | Table 2 |
| Risk of bias in studies | 18 | Present assessments of risk of bias for each included study. | Figure 3 |
| Results of individual studies | 19 | For all outcomes, present, for each study: (a) summary statistics for each group (where appropriate) and (b) an effect estimates and its precision (e.g. confidence/credible interval), ideally using structured tables or plots. | Figure 4, 5 and 6 |
| Results of syntheses | 20a | For each synthesis, briefly summarise the characteristics and risk of bias among contributing studies. | Figure 3 |
|  | 20b | Present results of all statistical syntheses conducted. If meta-analysis was done, present for each the summary estimate and its precision (e.g. confidence/credible interval) and measures of statistical heterogeneity. If comparing groups, describe the direction of the effect. | Figure 4, 5 and 6 |
|  | 20c | Present the results of all investigations of possible causes of heterogeneity among study results. | Page 6-10 |
|  | 20d | Present results of all sensitivity analyses conducted to assess the robustness of the synthesized results. | Page 11, 12 |
| Reporting biases | 21 | Present assessments of risk of bias due to missing results (arising from reporting biases) for each synthesis assessed. | Page 8 |
| Certainty of evidence | 22 | Present assessments of certainty (or confidence) in the body of evidence for each outcome assessed. | Page 11 |
| **DISCUSSION** | | |  |
| Discussion | 23a | Provide a general intrepretation of the results in the context of other evidence. | Page 12-14 |
|  | 23b | Discuss any limitations of the evidence included in the review. | Page 15 |
|  | 23c | Discuss any limitations of the review processes used. | Page 15 |
|  | 23d | Discuss implications of the results for practice, policy, and future research. | 10 |
| **OTHER INFORMATION** | | |  |
| Registration and protocol | 24a | Provide registration information for the review, including register name and registration number, or state that the review was not registered. | Page 15 |
|  | 24b | Indicate where the review protocol can be accessed, or state that a protocol was not prepared. | n.a |
|  | 24c | Describe and explain any amendments to information provided at registration or in the protocol. | n.a |
| Support | 25 | Describe sources of financial or non-financial support for the review, and the role of the funders or sponsors in the review. | Page 15 |
| Competing interests | 26 | Declare any competing interests of review authors. | Page 16 |
| Availability of data, code and other materials | 27 | Report which of the following are publicly available and where they can be found: template data collection forms; data extracted from included studies; data used for all analyses; analytic code; any other materials used in the review. | No template forms, extracted data, analytic code, or other materials are publicly available. |
